# Total knee Arthroplasty: risk factors for allogeneic blood transfusions in the South Asian population

**DOI:** 10.1186/s12891-017-1728-5

**Published:** 2017-08-23

**Authors:** Syed Hamza Mufarrih, Nada Qaisar Qureshi, Arif Ali, Azeem Tariq Malik, Huda Naim, Shahryar Noordin

**Affiliations:** 1 Aga Khan University, Karachi, Pakistan; 2 Dow International Medical College, Karachi, Pakistan; 3 Aga Khan University Hospital, Karachi, Pakistan

**Keywords:** Blood transfusion, Total knee Arthroplasty, Risk factors

## Abstract

**Background:**

Total knee arthroplasty (TKA) is the recommended treatment for end-stage knee osteoarthritis. Considering the various risks associated with intra and postoperative blood transfusions, better understanding is required with respect to the risk factors contributing to a greater possibility of blood transfusion during or after surgery. Although literature highlights several such factors, our study is among the first to identify these risk factors in the South Asian population which differs from other populations in several ways.

**Methods:**

The study consists of a review of 658 patients undergoing TKA from 2005 to 2015. Data was obtained from patient medical records and was analysed using logistic regression analysis. The relationship between each predictor and the outcome variable was calculated as an Odds ratio (OR), the threshold of significance for which was *p* = 0.25 and *p* = 0.05 for univariate and multivariable analysis respectively.

**Results:**

The mean age of the patient population was 63 years (78% female), 25% of whom received one or more blood transfusions. Multivariable analysis revealed 5 significant independent predictors for increased risk of blood transfusions including bilateral knee surgery (OR:5.51), preoperative anemia (OR:4.15), higher ASA (American Society of Anaesthesiologists) status (3–4) (OR:1.92), female sex (OR:3.44) and BMI (Body mass index) ≤30 (OR:1.79) while increasing co-morbidities and age (>60) were found to be insignificant.

**Conclusions:**

The factors identified for the South Asian population are largely similar to those for other populations. Identification of high risk patients will permit the application of an international multipronged approach which not only targets the modifiable risk factors but also the decision making process and blood management protocols in order to minimize the transfusion associated risks for a patient undergoing a TKA**.**

## Background

Total Knee Arthroplasty (TKA) is the treatment of choice for several end stage knee diseases. The aim of the procedure is to relieve pain and restore the mobility of the patient and has been proven to have excellent results [1]. Intra and post-operative blood transfusions increase the cost of surgery, not only directly but also through the use of staff time and hospital resources and prolonging hospital stay [2]. In developing countries, the chance of acquiring blood borne infections is an added risk associated with blood transfusions [3]. Previous literature identifies, estimated blood loss, preoperative anemia, bilateral procedures, advancing age, female sex, higher ASA (American Society of Anesthesiologists) status and low BMI (Body mass index) among others as significant predictors for blood transfusions following TKA [4–8]. To our knowledge, there has been no publication which assesses these risks in patients from South Asia. The genetic variation between different ethnicities has significant medical implications. The rationale for this is population-specific mutations, linkage disequilibrium and different selecting pressures [9]. This leads to the basis of unequal disease-associated and risk-associated allele distribution in different populations which results in discrete findings in different populations [9]. Similarly, literature highlights several other differences between populations worldwide for instance lower anti-coagulant requirements in Asian population suggests differences in the coagulation factors between different ethnicities [10–12] which would affect intra operative blood loss. South Asia also has one of the highest rates of anemia secondary to increased prevalence of carrier state of haemoglobinopathies and micronutrient deficiencies [13–17]. In addition to higher prevalence of anaemia, studies have also shown that Asians have a higher bleeding tendency [18]. While the rates of obesity in many Asian countries are similar to those in USA, the predisposition to developing obesity related non-communicable diseases such as osteoarthritis is higher is Asians [18, 19]. An important non-communicable disease prevalent in Asians is diabetes which alters haemostatic and thrombotic state of the body [20]. Along with this, ethnicity also plays a role when it comes to sociocultural beliefs regarding lifestyle modifications including dietary habits and physical activity along with poor accessibility to self-care facilities [21]. These factors, together with others such as a longer mean operative duration make it imperative to study blood transfusions intra and post-surgery in the Asian population. The aim of our study was to identify the factors which increase the risk of blood transfusions during and after total knee arthroplasty surgery in the South Asian population.

## Methods

### Study design and patient population

We conducted a retrospective review of the hospital course of 658 patients who underwent Total Knee Arthroplasty (TKA) between May 2005 and December 2015 at our University Hospital. All patients admitted for an elective TKA with a primary diagnosis of advanced osteoarthritis or rheumatoid arthritis refractory to conservative management were included in the study. Patients undergoing revision arthroplasty or having another orthopaedic procedure in addition to a TKA during one anaesthetic session, as well as patients with missing relevant clinical information were excluded from the study. Unilateral surgeries and staged bilateral procedures were included in the unilateral category while the simultaneous bilateral procedure patients were included in the bilateral category. Data was obtained from the medical record files of all patients included in the study. Data variables studied were patient demographics, body mass index (BMI), concomitant co-morbidities including Diabetes mellitus (DM), Hypertension (HTN), Asthma and chronic obstructive pulmonary disease (COPD), Thyroid Disease, Coronary artery disease (CAD), dyslipidaemia, Chronic kidney disease (CKD), preoperative and postoperative haemoglobin levels, length of hospital stay, type of anaesthesia, American Society of Anaesthesiologists (ASA) score, Anaesthesia and Operative time, estimated intraoperative blood loss, and any intra and post-operative blood transfusions received by the patient.

### Clinical course

Prior to the surgical procedure, every patient visited the orthopaedic clinic where a thorough evaluation and all preoperative investigations were done. The patients were also preoperatively evaluated by anaesthesia and relevant services depending on their co-morbidities in conjunction with anaesthesia. Patients received routine postoperative physiotherapy. Along with being vitally monitored, postoperative day one measurements of blood haemoglobin levels were requested to access the need for blood transfusions. There are 8 major societies who have published transfusion criteria. In general, the different guidelines have recommended that transfusion is not indicated for haemoglobin >10 g/dL, but the lower threshold varies from 6 g/dL to 8 g/dL [22, 23]. In our setup, patients were transfused blood after written consent, based on the criteria of either experiencing persistent symptoms such as dizziness, tachycardia, hypotension or shortness of breath with a haemoglobin level of less than 9 g/dl or asymptomatic anaemia with a blood haemoglobin level of less than 7 g/dl. Discharge was based on the attainment of a stable hemodynamic status and independent mobilization with support.

### Statistical analysis

Data analysis was performed using SPSS Version 22. Descriptive analyses were conducted on all study measures, including patient characteristics and features of their hospital stay, using measures of central tendency for continuous measures and proportions for binary measures. Before stating central tendencies, normality test was run on the data. For normally distributed data, mean was used as the measure of central tendency while median was used as the measure of central tendency for skewed data. The relationship between blood transfusion as the outcome variable with various predictors such as age, sex, type of surgery, ASA, number of co-morbidities, obesity, preoperative haemoglobin levels and operative time was analysed by fitting a logistic regression model. The outcome variable was treated as binary, by use of the following categories: no transfusions versus 1 or more transfusions. The threshold for statistical significance was set at *p* = 0.25 for univariate analysis and *p* = 0.05 for multivariable analyses. The variables found to be insignificant in univariate analysis were excluded from the multivariable analysis.

## Results

### Patient characteristics and features of hospital stay

The mean age of the patient population was 62.5 years with 78% female. Most patients had at least 1 but not more than 2 comorbid conditions, with hypertension and diabetes being the more prominent ones. The mean BMI of the patients was 30.95, categorized as obese in our study, supporting the well-established relationship between obesity and development and progression of osteoarthritis [24], which was also the most common diagnosis of the patients selected for surgery. More patients underwent simultaneous bilateral TKA (58.7%) in comparison to unilateral (41.3%) procedures. The mean preoperative hemoglobin level was in the normal range for females according to the cutoff value of >11, but was low for males (cutoff > 13.6). The mean operative duration for unilateral and bilateral surgeries was 146 and 257 min respectively. The co-morbidities found prevalent were hypertension (58.2%), diabetes (28.1%) and CAD (8.5%) among others. Most patients were diagnosed with Osteoarthritis (95.6%) while a small proportion suffered from other diseases such Rheumatoid arthritis (3.6%). The most common type of anesthesia used was General (45.5%), followed by epidural combined with general (34.4%) and epidural alone (20.1%). Out of the 658 patients, 25% received blood transfusions. Descriptive analysis for the above mentioned and additional measures are given in Tables 1 and 2.

**Table 1:** Patient Characteristics (continuous variables)

Variable	Central Tendency
Age (y)	62.5 ± 9.7
BMI (kg/m2)	30.47 ± 5.36
Pre-op Haemoglobin (g/dL)	
Male	13.16 ± 1.43
Female	12.11 ± 1.27
Operative Duration (min)	
Unilateral	143 ± 37
Bilateral	254 ± 56
Estimated Blood Loss (ml)	153 ± 124
Post-op Haemoglobin (g/dL)	
Male	11.14 ± 1.39
Female	10.17 ± 1.34
Length of Stay (days)	9.00 ± 3

**Table 2:** Patient Characteristics (non-continuous variables)

Variable	Categories	Percentage (%)
Gender	Male	21.9
	Female	78.1
No. of co-morbidities	0	29.8
	1–2	63.4
	≥3	6.84
ASA	Low risk (1–2)	77.5
	High risk (3–4)	22.5
Type of Surgery	Unilateral	41.3
	Bilateral	58.7
Transfusions	Received	25.1

### Effect of predictors on blood transfusions

The findings of our study have been tabulated in Table 3 as odds ratio (with 95% confidence interval) for univariate analysis and adjusted odds ratio (with 95% confidence interval) for multivariable analysis along with the significance of each finding as a *p*-value, the threshold for which is 0.25 for univariate analysis and 0.05 for multivariable analysis. Univariate analysis shows 6 significant variables for increased risk of blood transfusions: bilateral surgery (OR: 4.34), preoperative anaemia (OR: 2.20), higher ASA status (OR: 1.73), female gender (OR: 1.59), BMI ≤30 (OR: 1.37) and increasing number of co-morbidities (OR-1: 0.88, OR-2: 1.32, OR-3: 1.54, OR-4: 5.44). The effect of advancing age on the outcome variable was insignificant and was excluded from the multivariable analysis. Multivariable analysis revealed 5 significant variables increasing blood transfusions: bilateral surgery (OR: 5.51), preoperative anaemia (OR: 4.15), higher ASA status (OR: 1.92), female gender (OR: 3.44) and BMI ≤30 (OR: 1.79) while increasing number of co-morbidities was deemed insignificant.

**Table 3:** Risk factors for allogeneic blood transfusions

Variable	Odds Ratio (95% CI) Univariate analysis	*p*-value	Adjusted Odds Ratio (95% CI) Multivariate analysis	*p*-value
Type of Surgery		<0.001		<0.001
Unilateral	1		1	
Bilateral	4.34 (2.81, 6.69)		5.51 (3.34, 9.01)	
ASA		0.007		0.009
Low Risk (1–2)	1		1	
High Risk (3–4)	1.73 (1.16, 2.58)		1.92 (1.18, 3.14)	
Pre-op Haemoglobin		<0.001		<0.001
Normal	1		1	
Low	2.20 (1.52, 3.20)		4.15 (2.48, 6.96)	
Sex		0.049		<0.001
Male	1		1	
Female	1.59 (1.00, 2.52)		3.44 (1.84, 6.43)	
BMI		0.098		0.008
> 30 (Obese)	1		1	
≤ 30 (non-Obese)	1.37 (0.94, 2.00)		1.79 (1.17, 2.76)	
No. of co-morbidities		0.048		0.379
None	1		1	
1	0.88 (0.56, 1.38)		0.84 (0.49, 1.42)	
2	1.32 (0.82, 2.12)		1.06 (0.60, 1.87)	
3	1.54 (0.79, 2.99)		1.15 (0.51, 2.58)	
4	5.44 (1.25, 23.61)		4.10 (0.81, 20.77)	
Age		0.825		
< 60 years	1			
≥ 60 years	1.04 (0.72, 1.50)			

## Discussion

Our study identified bilateral Total knee arthroplasty surgery, preoperative anemia, the female sex, higher ASA grade (3–4) and lower BMI (≤30) as independent predictors for increased intra and post-operative blood transfusions. However, advancing age (>60) did not affect the outcome variable significantly. Increasing number of co-morbidities, although not an independent risk factor, also contributed to an increased risk of blood transfusions. These findings are mainly similar to those of previous studies done for other populations as discussed below except for the effect of increasing age. This encourages the use of international practices regarding blood loss and transfusion for the South Asian population despite the differences in genetics and lifestyle in comparison to other ethnicities.

Total Knee arthroplasty is now considered the gold standard for the treatment of end stage osteoarthritis [25]. With primary osteoarthritis being the most prevalent joint disease worldwide [26], it is crucial to study the factors affecting the outcomes of this surgery. While many studies have been done to study the long term outcomes of total knee arthroplasty surgery in terms of pain relief, restoration of mobility and overall quality of life [27, 28], lesser data is available on the improvement of the intra and postoperative course of the patient. Our study targets one aspect of the latter by studying the factors contributing to an increased risk of blood transfusion in patients undergoing Total Knee arthroplasty surgery.

Allogeneic blood transfusion can be a life-saving modality in the setting of acute blood loss as it is the fastest way to increase blood hemoglobin levels. Even though the process has evolved to much safer levels [29], statistics show the occurrence of side effects in 10% of the blood transfusions with 1/5000 transfusions suffering from serious adverse reactions, including hemolytic reactions, acute lung injury and multi organ failure secondary to bacterial contamination [30]. Additionally, allogeneic blood transfusions have been associated with an increased risk of complications including infections, fluid overload and an increased length of stay at the hospital [31, 32].

Our findings of simultaneous bilateral TKA being an independent risk factor blood transfusions is consistent with several findings in the past [2, 7, 33]. The greater amounts of blood loss may be due to simultaneous bilateral bone cuts and surgical trauma [5]. Research also shows that the second knee in bilateral sequential TKAs bleeds significantly more than the first one due to the hypothesized decrease in clotting factors following release of the first tourniquet and perioperative hypothermia as suggested by animal models and in vitro studies [34]. However, even with the benefit of decreased risk of blood transfusions in staged bilateral total knee arthroplasty (BTKA) (categorized as unilateral TKA in our study), there is evidence to suggest that the combined cost and duration of hospital stay together with the doubled risk of exposure to hospital environment and anesthesia for those undergoing staged BTKA overcome the advantage of requiring less blood transfusions. [35–38].

Results also revealed pre-operative hemoglobin to be a significant risk factor for allogeneic blood transfusions. These findings were consistent with numerous studies done in the past which have identified pre-operative hemoglobin as an independent risk factor for post-operative blood transfusions. [7, 39–46]. Because patients with low preoperative hemoglobin have a low reserve of red blood cells that are further lowered after the surgical procedure, they are at a higher risk of developing life-threatening anemia and therefore require blood transfusion to increase the hemoglobin level to an optimum level. Given this, correction of preoperative anemia by stimulating erythropoiesis through administration of iron and recombinant erythropoietin among other techniques has also been suggested [47–49].

High-risk patients (ASA > 3) were also at a higher risk of transfusions than low-risk (ASA 1–2) patients. Previous studies have agreed with this finding [8, 31, 39, 41, 50, 51]. This may be due to the association of ASA levels 3 and 4 with greater number of co-morbidities and a greater amount of intra operative blood loss [52].

Findings that females are at a higher risk of receiving blood transfusions have been supported by previous studies [2, 8]. Apart from the higher prevalence of iron deficiency anemia in women [53], this could be due to having the same cutoff value of 9 g/dl as the criteria for blood transfusion for both sexes while having a normal range of hemoglobin lower than that of men.

Even though most studies show no correlation between BMI and risk of blood transfusions [43–46, 54], few studies have stated that a lower BMI (<30) serves as an independent factor for increased risk of blood transfusions in comparison to obese individuals who have larger volume and a lower percentage of estimated blood loss (EBL) volume [39–42, 55]. Our findings agree with this as the results show that a BMI ≤ 30 acts as an independent factor for increased risk of blood transfusions.

Our study also identifies increasing number of comorbid conditions to increase the risk of blood transfusions, although not independently. We did not find any studies which have made the same comparison. Our findings may be due the association of comorbid conditions with higher ASA status which has been shown to be an independent predictor for blood transfusions.

Previous studies have shown advancing age (>60) to be a significant predictor of increased risk of blood transfusions following TKA surgery [2, 7, 8, 45, 51]. Diminished postoperative hematopoietic regenerative capacity secondary to advancing age could account for these findings [56]. Our findings do not demonstrate this relationship. This may be due to the fact that the mean age of the population being studied was 62.5 years, thus decreasing the sensitivity of our study to the effects of advancing age (≥60).

Having stated the risks associated with blood transfusions and the factors contributing to increased chances of receiving them, it is crucial to apply methods of minimizing their occurrence during the intra and postoperative course of the patient’s experience. A multipronged approach can be adopted to target not only the various risk factors, but also the decision making process and blood management protocols following identification of high risk patients [57].

### Caveats

As a single centre study, the number of patients studied is not the true representation for the said population, which is the primary focus of our study. To our knowledge, this research is the first to study the South Asian population which differs from other populations in several ways, as discussed earlier. For greater accuracy and generalizability, multi-centred studies should be carried out including hospitals from several countries of South Asia.

## Conclusions

Our study identifies simultaneous bilateral TKA surgery, preoperative anaemia, higher ASA grade (3 and above), female gender, and lower BMI (≤30) as independent predictors for an increased risk of blood transfusions during and following knee arthroplasty surgery in South Asian patients. These factors are similar to those for other populations thus encouraging the use of international guidelines for blood management protocols despite the differences in the genetic makeup and lifestyle between South Asians and other populations. This information will help us in identification of high risk patients and correction of modifiable risk factors to minimize the need for blood transfusions in South Asian patients undergoing TKA.

## Abbreviations

ASA: American society of anaesthesiologists
BMI: Body Mass Index
BTKA: Bilateral total knee arthroplasty
CAD: Coronary artery disease
CI: Confidence interval
CKD: Chronic kidney disease
OR: Odds ratio
COPD: Chronic obstructive pulmonary disease
DM: Diabetes mellitus
EBL: Estimated blood loss
HTN: Hypertension
TKA: Total knee arthroplasty
